# Loop-mediated Isothermal Amplification-Single Nucleotide Polymorphism Analysis for Detection and Differentiation of Wild-type and Vaccine Strains of Mink Enteritis Virus

**DOI:** 10.1038/s41598-018-26717-6

**Published:** 2018-05-30

**Authors:** Peng Lin, Honglin Wang, Yuening Cheng, Shanshan Song, Yaru Sun, Miao Zhang, Li Guo, Li Yi, Mingwei Tong, Zhigang Cao, Shuang Li, Shipeng Cheng, Jianke Wang

**Affiliations:** 1 Key Laboratory of Special Animal Epidemic Disease, Ministry of Agriculture, Changchun, 130112 People’s Republic of China; 2 Institute of Special Animal and Plant Sciences, Chinese Academy of Agricultural Sciences, Changchun, 130112 People’s Republic of China; 3 Shandong Sinder Technology Co., Ltd, Zhucheng, Shandong 262204 People’s Republic of China

## Abstract

Broad coverage of mink enteritis virus (MEV) vaccination program in northeast of China has provided effective protection from mink viral enteritis. Nevertheless, MEV vaccine failures were reported due to continually evolving and changing virulence of field variants or wild-type MEV. In this study, a combined loop-mediated isothermal amplification (LAMP) and single nucleotide polymorphism (SNP) method, named LAMP-SNP assay, was developed for detection and differentiation of wild-type and vaccine strains of MEV. Four primers in MEV-*VP2*-LAMP were used to detect both wild-type and vaccine strains of MEV in our previous publication, and other four primers in LAMP-SNP were designed to amplify the *NS1* gene in wild-type MEV and only used to detect wild-type viruses. The LAMP-SNP assay was performed in a water bath held at a constant temperature of 65 °C for 60 min. LAMP-SNP amplification can be judged by both electrophoresis and visual assessment with the unaided eyes. In comparison with virus isolation as the gold standard in testing 171 mink samples, the percentage of agreement and relative sensitivity and specificity of the LAMP-SNP assay were 97.1, 100%, and 94.0%, respectively. There were no cross-reactions with other mink viruses. The LAMP-SNP assay was found to be a rapid, reliable and low-cost method to differentiate MEV vaccine and field variant strains.

## Introduction

Mink viral enteritis (MVE) is an acute hemorrhagic enteritis that affects *Mustela lutreola* and *Neovison vison* of different ages, was first discovered in 1949, and mink enteritis virus (MEV) is a member of the *Parvoviridae* family^1,2^. Newborn and juvenile minks are particularly susceptible to MEV, and MVE affects the mink industry worldwide^3–6^. The main symptoms of MVE are severe diarrhea, feces containing sloughed-off gray intestinal mucosal cells, and severe leukopenia^7^. MEV is highly transmissible and can cause a fatality rate up to 80%^1^, which is highly detrimental to the mink economy^8,9^. Currently, vaccination of all farmed minks with the MEV vaccine has well controlled the MEV infection, however, the high rate of mutation of MEV (~10^−4^ substitutions per site per year^10^) can lead to new-variant strains and MVE epidemics. Although widespread vaccination against MVE has been executed for six decades, sometimes there were vaccine failure in minks and vaccinated animals showed clinical symptoms of MVE infections. Rapid and accurate diagnostic methods for the detection and differentiation of MEV wild-type and vaccine strains are required for effective control of this devastating mink disease.

MEV is a non-enveloped virus containing a ~5 kb ssDNA genome within an icosahedral capsid of 18–24 nm. The genome contains two large open reading frames (ORFs), which give rise to smaller structural ORFs by alternative splicing^5^. The most common methods currently in use for MEV detection include PCR, virus isolation, electron microscopy, and serological methods (Hemagglutination and Hemagglutination inhibition assays), which are effective in MEV diagnostics but are unable to differentiate between MEV wild-type and vaccine strains. To address the MEV strain differentiation need, we combined loop-mediated isothermal amplification (LAMP) and single nucleotide polymorphism (SNP) technologies in a LAMP-SNP assay. LAMP is a new isothermal amplification technology that performs auto-cycling strand-displacement DNA synthesis with *Bacillus stearothermophilus* (*Bst*) DNA polymerase at constant temperature^11,12^. Four or six primers for specific target genes are used to produce amplification products with stem-loop structures^13^ in approximately one hour without the denaturation step. In addition, specific LAMP assay could be highly sensitive and specific^14^. LAMP is an easy and time-saving test^15^, and the cost is low because LAMP does not require the expensive PCR reagents or sophisticated equipment. LAMP has great potential to be used widely to detect pathogens and biological markers^15–17^.

In this study, we aimed to detect and differentiate wild-type and vaccine MEV strains from immunized minks. The forward inner primer (FIP) and the backward inner primer (BIP) were designed to contain an SNP at nucleotide 1846 of the *NS1* gene (G for wild-type strains and A for vaccine strain). When using primers for the wild-type strains, DNA synthesis from a stem-loop structure and LAMP amplification cycling continues with the wild-type strains, but not for the vaccine strain. Therefore, the LAMP-SNP method can effectively distinguish between wild-type and vaccine strains without complicated DNA sequencing. This system provides a relatively quick and easy method for an on-site diagnostic test of MEV pathogens.

## Results

### Development of the LAMP-SNP assay

**Figure 1:**
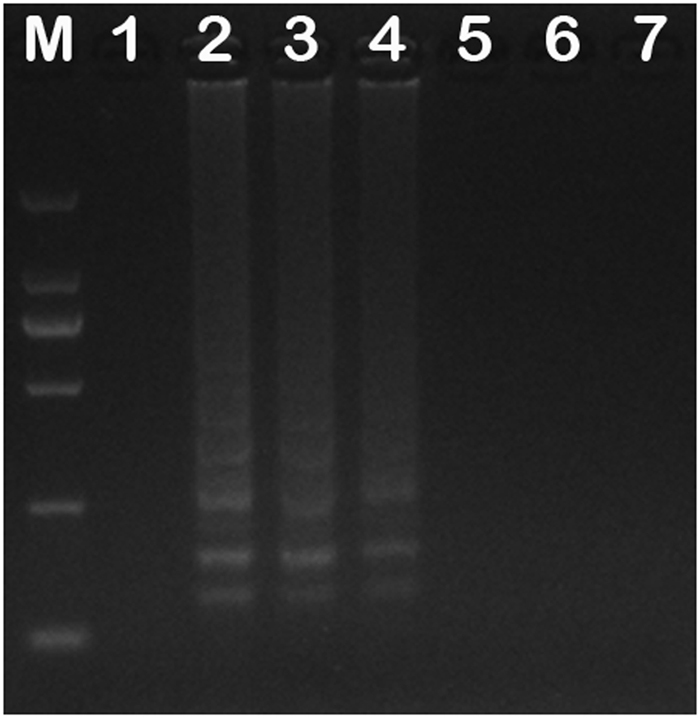
Detection MEV and non-MEV viruses by the LAMP-SNP assay. Lane M: DNA Marker DL2000 (TaKaRa, Dalian, China). Lane 1: LAMP amplification products with MEVB DNA as the template; Lanes 2–4: LAMP amplification products with MEV SD, MEV-Z6, and MEV-LN10 DNAs as templates, respectively; Lanes 5–7: LAMP amplification products with DNA/cDNAs of MPRV-J, AMDV-G, and CDV3 as templates, respectively. Full-length gel is presented in Supplementary Fig. 1.

Total DNA was extracted from each of suspected MEV samples, non-MEV strains, wild-type MEV strains, and the vaccine strain MEVB. The MEV *VP2* gene LAMP assay (MEV-*VP2-*LAMP) detected MEV as observed by the presence of typical ladder-like bands representing several inverted repeat LAMP products upon 2.0% agarose gel electrophoresis (data not shown). The MEV-*NS1*-LAMP (LAMP-SNP) assay detected wild-type MEV strains, including MEV SD, MEV-Z6, and MEV LN10 strains, but not the vaccine strain MEVB or the non-MEV viruses, mink pseudorabies virus (MPRV) J strain, Aleutian mink disease virus (AMDV) G strain, and canine distemper virus (CDV) 3 strain, because they did not contain the inverted repeat structures required for amplification (Fig. 1).

**Figure 2:**
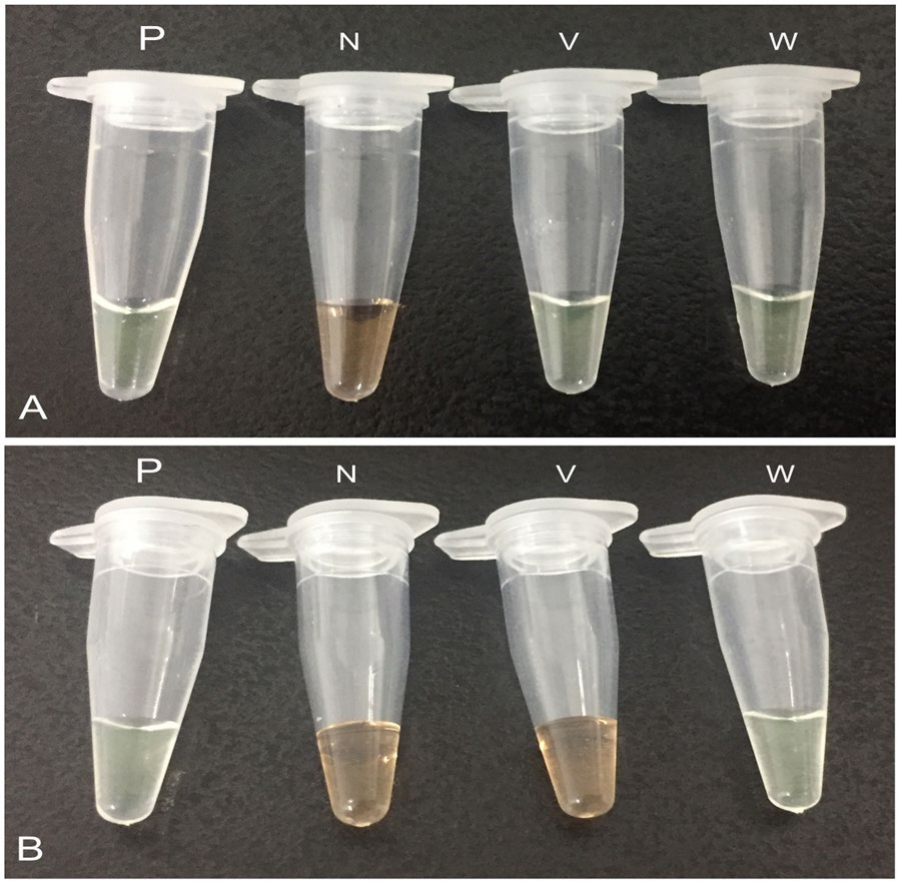
Visual inspection LAMP products by using SYBR Green I. (**A**) P: positive control (MEVB), N: negative control (double distilled water), V: vaccine MEVB strain, W: wild-type MEV SD isolate. (**B**) P: positive control (MEV SD), N: negative control (double distilled water), V: vaccine MEVB strain, W: wild-type MEV SD isolate. For visual inspection of the LAMP products, 4 μL diluted SYBR Green I dye 10,000× concentration in double distilled water was added to the reaction tube after the reaction termination. Yellowish green and reddish orange represent positive and negative reactions, respectively. Full-length tubes are presented in Supplementary Fig. 2A,B.

Moreover, LAMP-SNP products can be detected not only using agarose gel electrophoresis but also by visual observation of color change with intercalator SYBR Green I. In the MEV-VP2-LAMP assay, upon addition of the SYBR green I dye to tubes after the LAMP reaction, the color changed to yellowish green both vaccine and wild-type MEV virus and positive control, while that remained reddish orange in the negative control. And in the LAMP-SNP assay, the color of tube of wild-type MEV virus was changed to yellowish green and that of vaccine virus remained reddish orange (Fig. 2). With the two assays, we can simultaneously detect and differentiate wild-type and vaccine strains of MEV.

### Detection limit of the LAMP-SNP assay

**Figure 3:**
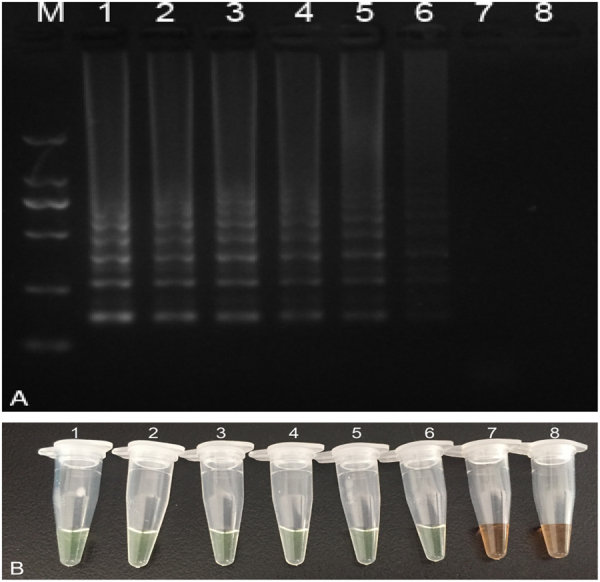
Detection limit of LAMP-SNP assay. (**A**) Electrophoresis. Lane M: DNA Marker DL2000 (TaKaRa, Dalian, China). Lanes 1–8 represent 2 × 10^6^–2 × 10^−1^ copies/mL of pT-MEV-NS1 plasmid, respectively. (**B**) Visual inspection. Tubes 1–8 represent 2 × 10^6^–2 × 10^−1^ copies/mL of pT-MEV-NS1 plasmid, respectively. Both methods exhibited the same results, the detection limit was 10^1^ copies/mL, with no differences between electrophoresis and visual inspection. Full-length gels and tubes are presented in Supplementary Fig. 3A,B.

To determine the detection limit of the LAMP-SNP assay, the plasmid pT-MEV-NS1 was 10-fold serially diluted and used as the template. The lower limit of detection of LAMP-SNP assay was 10^1^ copies of pT-MEV-NS1 per reaction. The detection was determined not only using agarose gel electrophoresis but also by visual judgment by the unaided eye with intercalator SYBR Green I (Fig. 3).

### Specificity and Sensitivity of the LAMP-SNP assay

**Figure 4:**
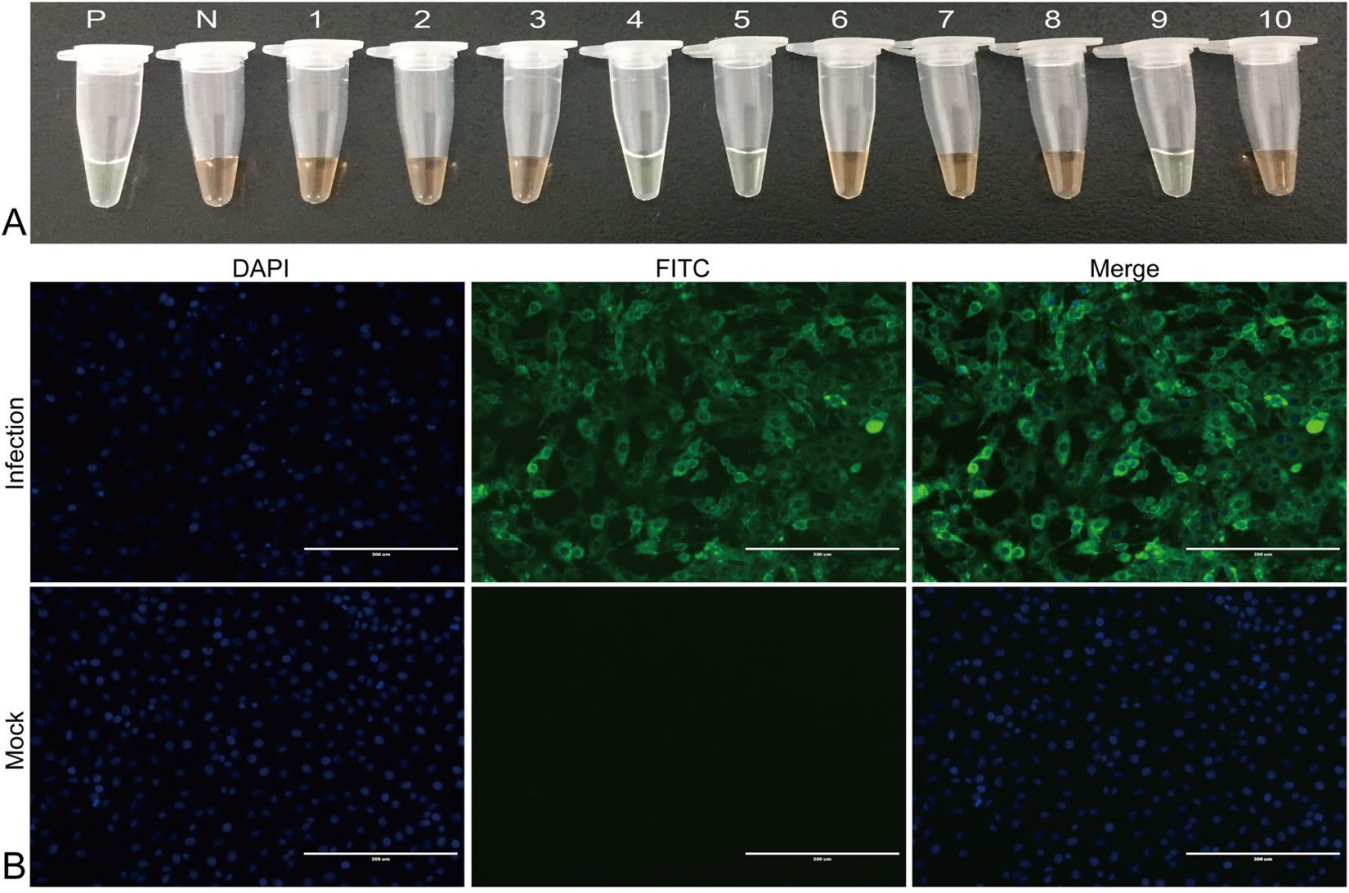
Evaluation of clinical specimens by (**A**) LAMP-SNP and (**B**) virus isolation. (**A**) P: positive control (MEV SD), N: negative control (double distilled water), tubes 1–10 represent different clinical fecal samples. (**B**) Virus isolation (MEV-Z6) was identified by an indirect immunofluorescence assay using a monoclonal antibody against the VP2 protein of MEV and goat anti-mouse IgG-FITC conjugated secondary antibody. Specific immunofluorescent signals were detected in cells exposed to the respective tissue filtrates but not in mock-infected control cells. Full-length tubes and immunofluorescence pictures are presented in Supplementary Fig. 4A, B.

Of the 171 mink specimens tested by both virus isolation and LAMP-SNP assay, 88 were positive for MEV and 78 were negative for MEV in both tests, the remaining 5 samples were negative by virus isolation but positive by LAMP-SNP assay. Therefore, this new LAMP-SNP assay for MEV achieved 100% (88/88) of sensitivity and 94.0% (78/83) of specificity (Table 1). The 88 positive samples were identified as wild-type by LAMP-SNP assay and they were also identified as wild-type virus by DNA sequencing. Figure 4 showed detection of clinical specimens by LAMP-SNP assay and indirect immunofluorescence assay. This new LAMP-SNP assay for MEV showed no cross-reactions with the other mink viruses tested, including MPRV, AMDV, and CDV (Fig. 1).

**Table 1:** Comparison of the sensitivity and specificity of LAMP-SNP and virus isolation for detection of MEV in 171 samples.

LAMP-SNP (MEV-*NS1*-LAMP)	Virus isolation		
	Positive	Negative	Total
Positive	88	5	93
Negative	0	78	78
Total	88	83	171

Percentage of agreement: (88 + 78)/171 = 97.1%; relative sensitivity: 88/88 = 100%;

relative specificity: 78/83 = 94.0%.

## Discussion

MVE, which is caused by MEV, is an acute infectious disease that threatens mink farming industry^18^. Currently, vaccination with attenuated and inactivated vaccines is the primary method to prevent MVE. Inoculation with an attenuated vaccine provides sustained immune responses, but the vaccine lacks stability and the attenuated virus can recover pathogenicity. Inactivated vaccines are easy to mass produce, but they may be less effective than attenuated vaccines and requires repeated inoculations. MVE prevention in China was primarily carried out by immunizations with the inactivated MEV vaccine, however, clinical symptoms, including diarrhea, marasmus, and death, occasionally occurred in minks after inoculation. Therefore, it was important to determine whether the symptoms resulted from the vaccine preparation or whether the minks were infected with wild-type MEV prior to inoculation.

The LAMP assay, which amplifies nucleic acids *in vitro*, detects antigens simply and rapidly and does not require sophisticated equipment^19^. In this study, we found that SNP typing using the LAMP assay is highly specific and allows for amplification of specific alleles. In this method, both strands of DNA containing a specific SNP are amplified simultaneously with calibration. If the SNP is present on both strands, amplification occurs, thus SNPs polymorphic typing is achieved by detecting the presence or absence of amplification reactions. We successfully developed a diagnostic system that combines LAMP and SNP technologies to detect wild-type MEV in mink that were vaccinated against MEV. This diagnostic system has potential use for laboratory and clinical diagnosis practices.

There are many methods for the detection and diagnosis of MEV, including virus isolation, the hemagglutination assay, electron microscopy, ELISA, and PCR^3^, but they have disadvantages, and cannot differentiate between wild-type and vaccine strains of MEV around the world. A disadvantage of all of these diagnostic methods is that they require expensive instruments and special material, which limits their clinical applications, especially for field-testing. In contrast, LAMP is a simple and efficient method that only requires a constant temperature instrument for incubation under isothermal conditions, thus this method can be used for quick diagnosis of MVE and is suitable for field-testing.

We found that our LAMP-SNP assay diagnosed specifically different types of MEV. In addition, the LAMP-SNP assay was more sensitive than virus isolation and did not require an additional gene-sequencing step. This novel detection technique is useful for timely clinical diagnosis of MEV so that the infection can be controlled. In conclusion, the LAMP-SNP assay is rapid, simple, sensitive, and specific and can rapidly detect and differentiate between wild-type and vaccine MEV strains.

## Methods

### Viruses

The MEVB^20^ was a vaccine strain from the Jilin Teyan Biological Technology Company, Changchun, China; the wild-type viruses, MEV SD^21^, MEV-Z6^22^, and MEV-LN10^23^, were isolated from the Shandong, Jilin, and Liaoning provinces in China. The non-MEV viruses, including MPRV-J, AMDV-G^24^, and CDV3^25^, were maintained at the Key Laboratory of Special Animal Epidemic Disease, Ministry of Agriculture, PR China, Institute of Special Animal and Plant Sciences, Chinese Academy of Agricultural Sciences. All the viruses were prepared as described in some previous studies^20–25^.

### Primer design

**Figure 5:**
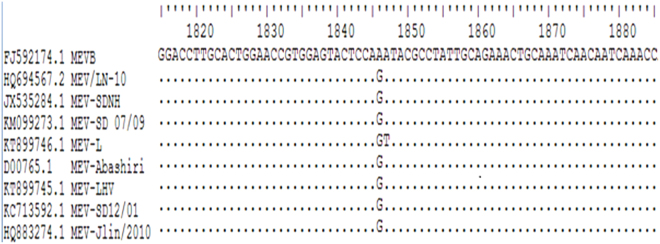
Partial sequence alignment of *NS1*. *NS1* sequences from wild-type strains and vaccine strain MEVB were analyzed. In wild-type strains, there is a G at position 1846 of *NS1*, whereas there is an A at that position in the vaccine strain. This nucleotide difference was used to design the differentiation primers used in the LAMP-SNP assay.

**Figure 6:**
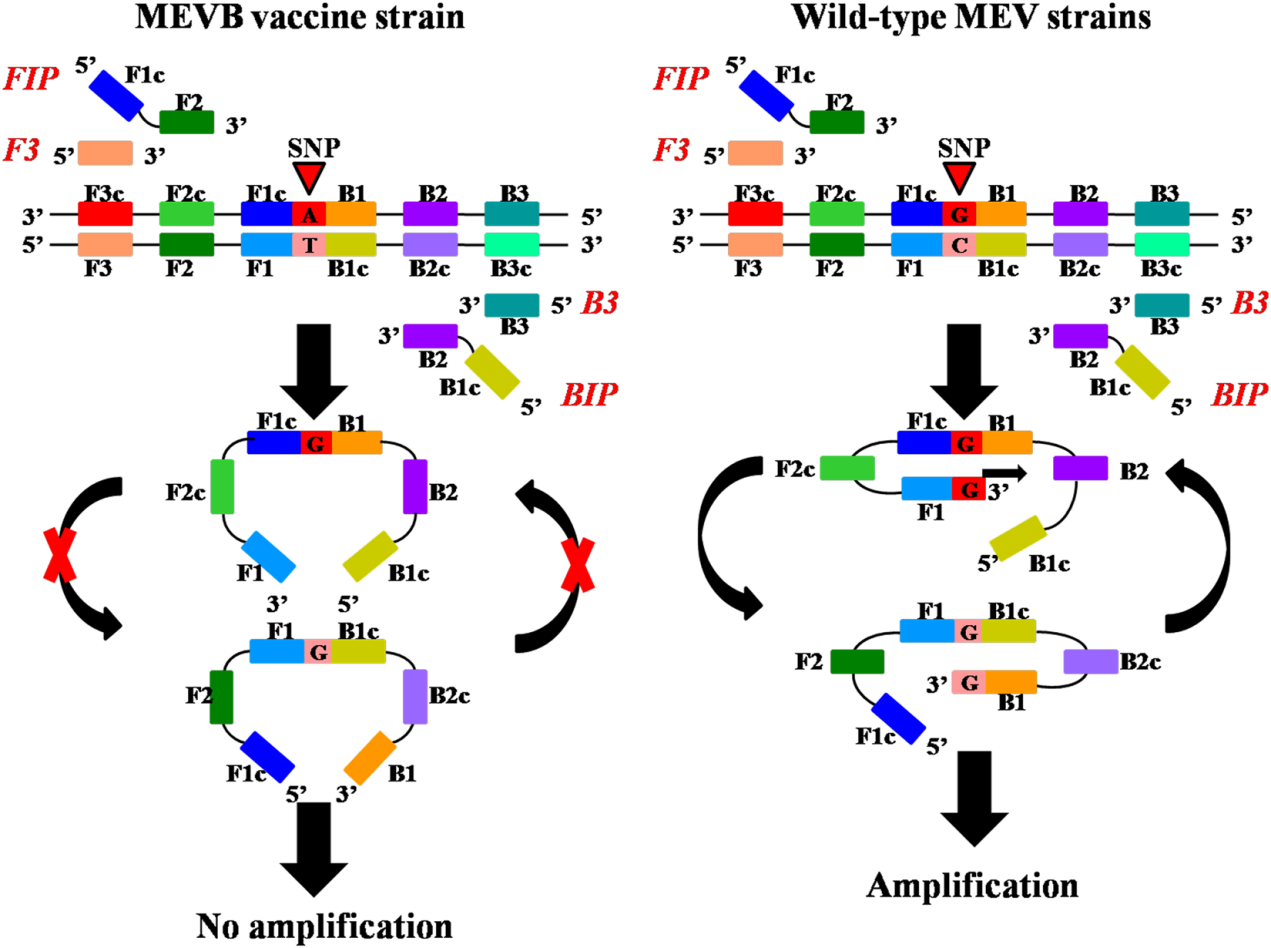
Schematic of single nucleotide polymorphism typing using the LAMP method. The BIP and FIP primers were designed to contain the wild-type SNP allele. With wild-type DNA template and these primers, a dumbbell-like structure is formed and amplification occurs. If the template DNA is from the vaccine strain MEVB or from a non-MEV virus, a dumbbell-like structure will not form and amplification will not occur.

The primers used in the MEV-VP2-LAMP assay to detect MEV were designed in a previous study^15^. The primers F3-NS1, B3-NS1, FIP-NS1, and BIP-NS1 were designed using online LAMP primer software PrimerExplorer (http://primerexplorer.jp/e/), and the primer sequences are shown in Table 2. Primers to *NS1* gene from wild-type MEV strains and the vaccine strain MEVB were aligned using BioEdit software (Fig. 5). The nucleotide at position 1846 of the *NS1* gene of wild-type strains is G, while that is A for vaccine strain. The alignment showed that the primers for the wild-type MEV strains were conserved, but that the primers for the vaccine strain MEVB were not. The primer design principle is shown in Fig. 6.

**Table 2:** Primers were used in this study for PCR, MEV-*VP2*-LAMP, and LAMP-SNP (MEV-*NS1*-LAMP).

Assays	Primers	Primer Sequence (5′-3′)	Source
pT-MEV-NS1 plasmid construction	NS1 F	CGCGGATCCATGTCTGGCAACCAGTATA	This study
	NS1 R	CGGCTGCAGTTAATCCAAGTCGTCTCGAA	
MEV-*VP2*-LAMP	VP2 F3	GTAAACCATGTAGACTAACACA	ref.^15^
	VP2 B3	AACCTCAGCTGGTCTCAT	
	VP2 FIP (F1c + F2)	TCCTTCAGATTGAGGCAAAGAATTT + TACATGGCAAACAAATAGAGC	
	VP2 BIP (B1c + B2)	AGGAGTTCAACAAGATAAAAGACGT AGTAGCTTCAGTAATATAGTCTGT	
LAMP-SNP (MEV-*NS1*-LAMP)	NS1 F3	TAGAGACACAAGCGGCAA	This study
	NS1 B3	CCTCTATTTCGGACCACG	
	NS1 FIP (F1c + F2)	CTGGAGTACTCCACGGTTCC TCCTCAGAGTCAAGACCA	
	NS1 BIP (B1c + B2)	GATACGCCTATTGCAGAAACT TTGCACGTCTTTGTGAGT

### Collection of mink samples

A total of 171 mink samples, including 67 mink tissue specimen and 104 mink fecal samples, were collected in our lab. These tissues were the homogenates of mink intestines and the fecal samples (Supplementary Table 1) were collected with sterile swabs, diluted in PBS, and stored at −20 °C until further use. All the samples were from Hebei, Shandong, Jilin, Liaoning and Heilongjiang in China, and all the animals show clinical symptoms of MVE include anorexia, vomiting, and diarrhea. MEVB, MEV SD, MEV-Z6, and MEV-LN10 strains were cultured in F81 cells^26^.

### DNA extraction

Total DNA was extracted from fecal samples, cultured cells infected with MEV, and cultured cell that were not infected with MEV using the Takara MiniBEST Viral RNA/DNA Extraction Kit Ver. 5.0 (Takara Biotechnology Co., Ltd., Dalian, China) according to the manufacturer’s instructions. The extracted DNAs were used as the templates in the LAMP assays.

### pT-MEV-NS1 plasmid construction

MEV-NS1 forward and reverse primers (NS1 F and NS1 R, see Table 2) were used to amplify *NS1* from MEV-SD. The *NS1* PCR product was cloned into the pMD18-T vector (Takara Biotechnology Co., Ltd., Dalian, China) and the resulting plasmid was named pT-MEV-NS1 and used as the positive control template.

### Detection and differentiation of wild-type and vaccine MEV strains by LAMP-SNP assay

Both wild-type and vaccine viruses were detected by MEV-*VP2*-LAMP assay^15^. LAMP-SNP reactions were performed in a total volume of 25 μL and contained 0.4 μL (10 pM) each of the F3 and B3 primers, 2.5 μL (10 pM) each of the FIP and BIP primers, 3.5 μL (10 mM) of dNTPs, 2.5 μL of 10 × *Bst* DNA polymerase buffer, 0.5 μL (8 U) of *Bst* DNA polymerase (New England BioLabs, Herts, UK), 2 μL of template DNA, 4 μL (100 mM) of MgSO_4_, and 6 μL (20 μM) of betaine. The amplifications were performed at 65 °C in water bath for 60 min and were terminated by incubating the reactions at 80 °C in water bath for 10 min. The LAMP amplification products were analyzed by 2.0% (w/v) agarose gel electrophoresis. Besides agarose gel electrophoresis, the products of LAMP-SNP were judged by naked eyes with the addition of SYBR Green I (Solarbio, Beijing, China) dye into the tubes. Upon addition of the SYBR green I dye to tubes after the LAMP-SNP reaction, the color changed to yellowish green in the positive reactions and remained reddish orange in a negative reaction.

### Detection limit of the LAMP-SNP assay

The detection limit of the LAMP-SNP assay was assessed by using a panel of 10-fold serial dilutions of pT-MEV-NS1 (2 × 10^6^–2 × 10^−1^ copies/mL). The plasmid dilution series was used in the LAMP-SNP assay to determine the minimum concentrations required for detection by the assay.

### Specificity and Sensitivity of the LAMP-SNP assay

A total of 171 mink tissue specimen, consisting of 88 positives and 83 negatives for MEV, were diagnosed by virus isolation for the “gold standard” and used for the LAMP-SNP assay’s specificity and sensitivity evaluation test to detect MEV from clinical mink specimen. All the specimens were collected from healthy minks or wild-type strains MEV SD, MEV Z6, and MEV-LN10. The cross-reaction of the LAMP-SNP assay was evaluated with other mink viruses including MPRV, AMDV, and CDV.

Virus isolation was performed using F81 cells as described previously^15^. And the virus isolation was identified by indirect immunofluorescence assay as follows: F81 cells were infected with MEV isolates at an input MOI of 0.01 TCID_50_ per cell. Following 48 h of incubation, cells were immunostained using a monoclonal antibody specific for the VP2 protein of MEV^27^ and FITC-labeled goat anti-mouse IgG. Nuclei were stained with 40,6-diamidino-2-phenylindole dihydrochloride (DAPI) (Sigma).

### Ethics in publishing

The methods were carried out in accordance with Animal Epidemic Prevention Law of the People’s Republic of China^28^. Animal experiments were performed in accordance with animal ethics guidelines^28^ and approved by the Institute of Special Animal and Plant Sciences of the Chinese Academy of Agricultural Sciences.

## Electronic supplementary material

Supplementary information of full figures

Supplementary table 1
