# Supplementary material for: Loop-mediated Isothermal Amplification-Single Nucleotide Polymorphism Analysis for Detection and Differentiation of Wild-type and Vaccine Strains of Mink Enteritis Virus
Source: Sci Rep. 2018 May 30;8:8393. doi: 10.1038/s41598-018-26717-6 (PMC5976767; doi:10.1038/s41598-018-26717-6)
Supplement: Supplementary file 1 — Supplementary information of full figures [file 41598_2018_26717_MOESM1_ESM.pdf]

Loop-mediated Isothermal Amplification-Single Nucleotide Polymorphism Analysis for Detection and Differentiation of Wild-type and Vaccine Strains of Mink Enteritis Virus

Peng Lin[1, *], Honglin Wang[2, *], Yuening Cheng[1], Shanshan Song[1], Yaru Sun[1], Miao Zhang[1], Li Guo[1], Li Yi[1], Mingwei Tong[1], Zhigang Cao[1], Shuang Li[1], Shipeng Cheng[1] & Jianke Wang[1]

[1]Key Laboratory of Special Animal Epidemic Disease, Ministry of Agriculture, P.R. China; Institute of Special Animal and Plant Sciences, Chinese Academy of Agricultural Sciences, Changchun 130112, People's Republic of China

[2]Shandong Sinder Technology Co., Ltd， Zhucheng, Shandong 262204, People's Republic of China

Key Laboratory of Special Animal Epidemic Disease, Ministry of Agriculture, P.R. China; Institute of Special Animal and Plant Sciences, Chinese Academy of Agricultural Sciences, No. 4899, Juye Street, Jingyue District, Changchun, People's Republic of China 130112.

*These authors contributed equally to this work.

Tel.: +86 431 81919845; Fax: +86 431 81919800.

Correspondence and requests for materials should be addressed to J.-K.W. (email: tcswjk@126.com)

Legends:

Supplementary Figure 1: Full-length gel of figure 1;

Supplementary Figure 2A and 2B: Full-length tubes of figure 2;

Supplementary Figure 3A and 3B: Full-length gels and tubes of figure 3;

Supplementary Figure 4A and 4B: Full-length tubes and immunofluorescence pictures of figure 4.

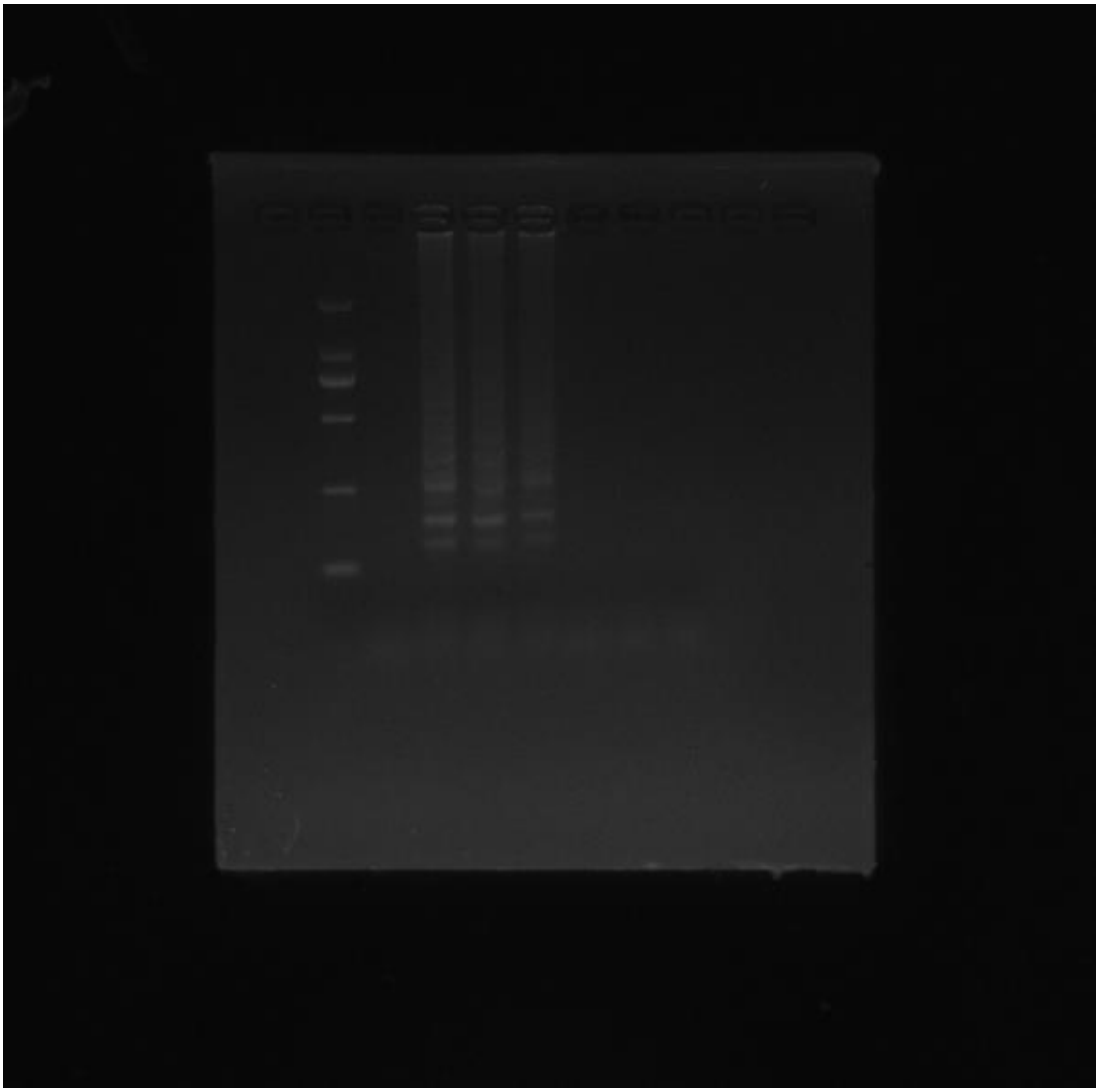

Supplementary Figure 1

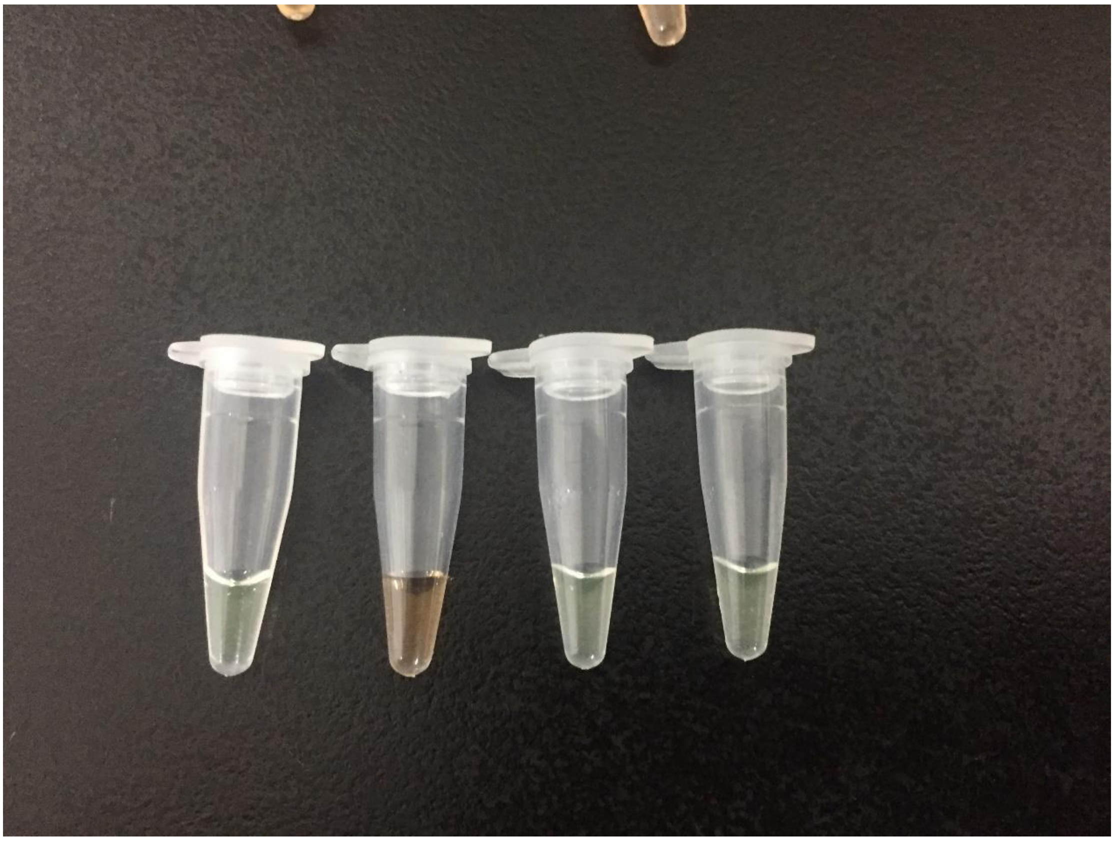

Supplementary Figure 2A

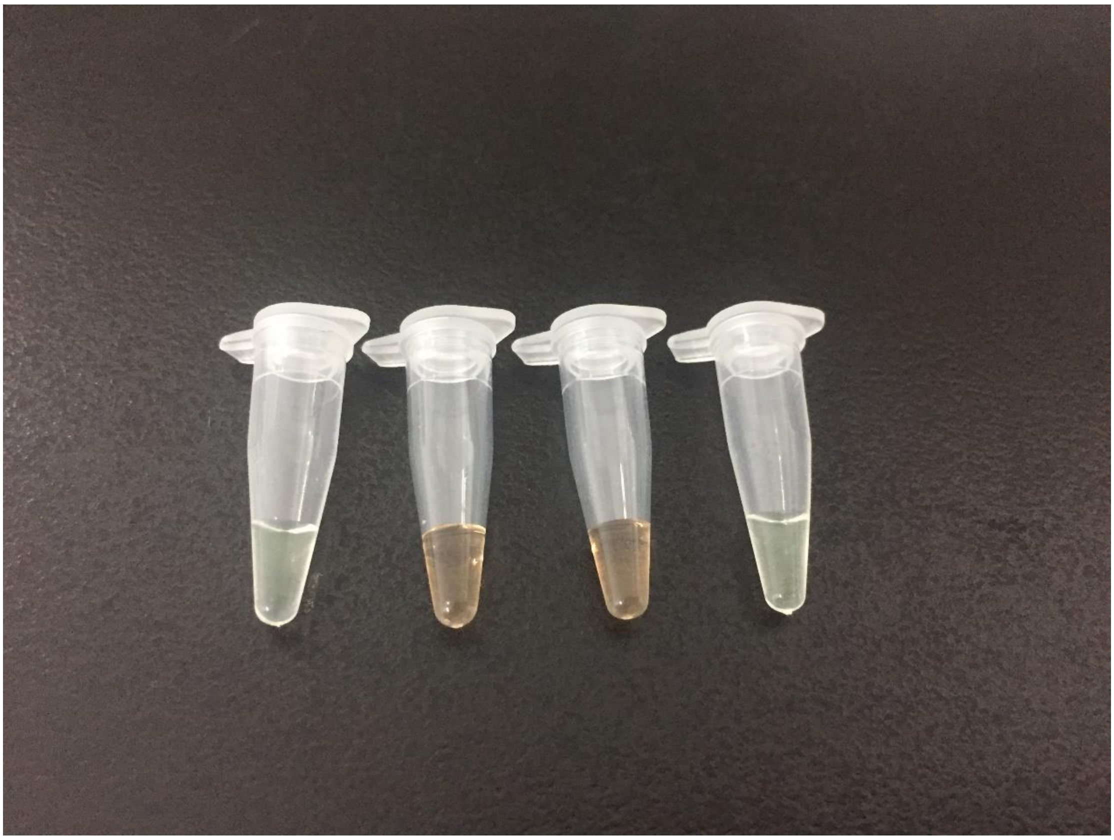

Supplementary Figure 2B

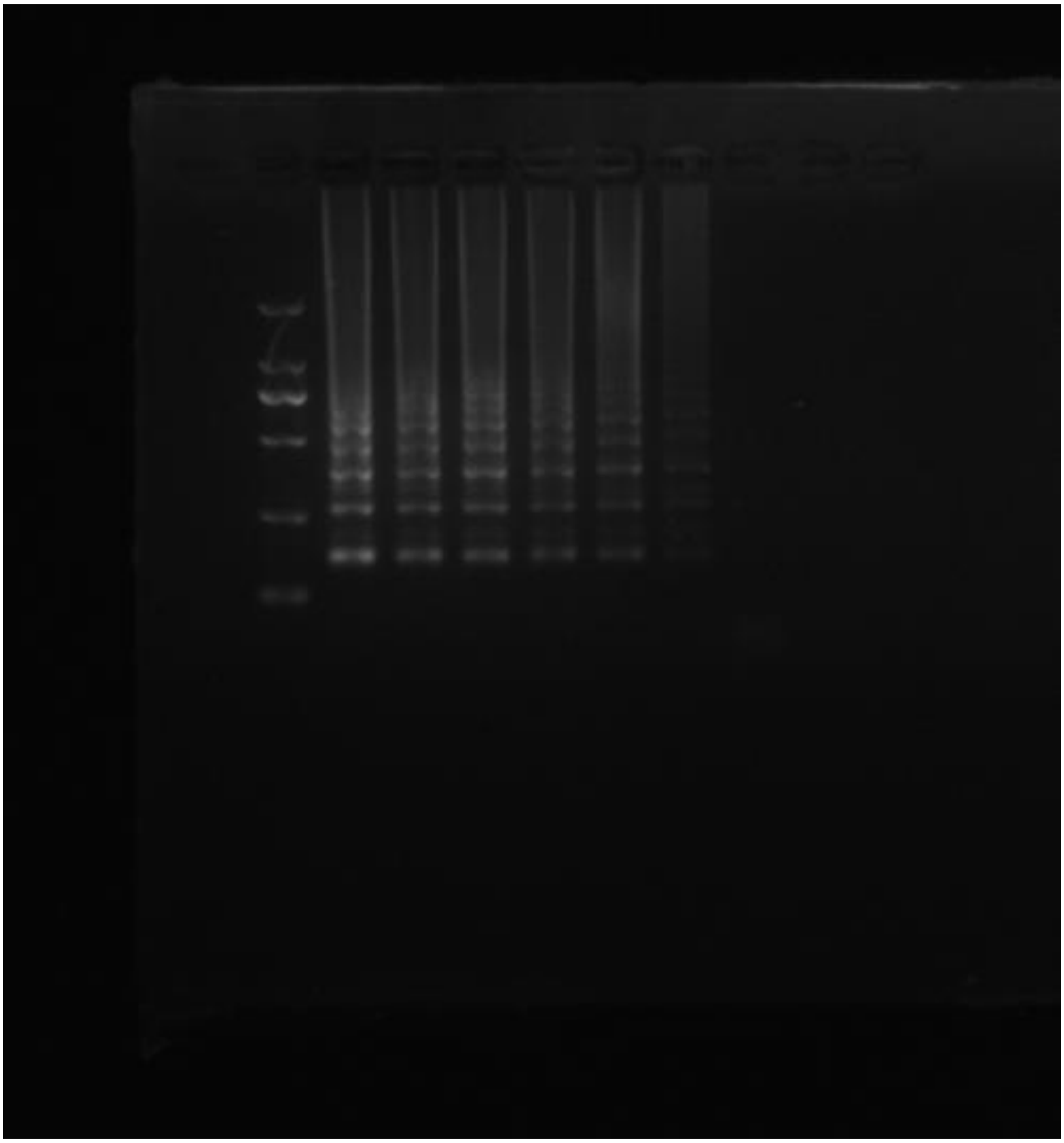

Supplementary Figure 3A

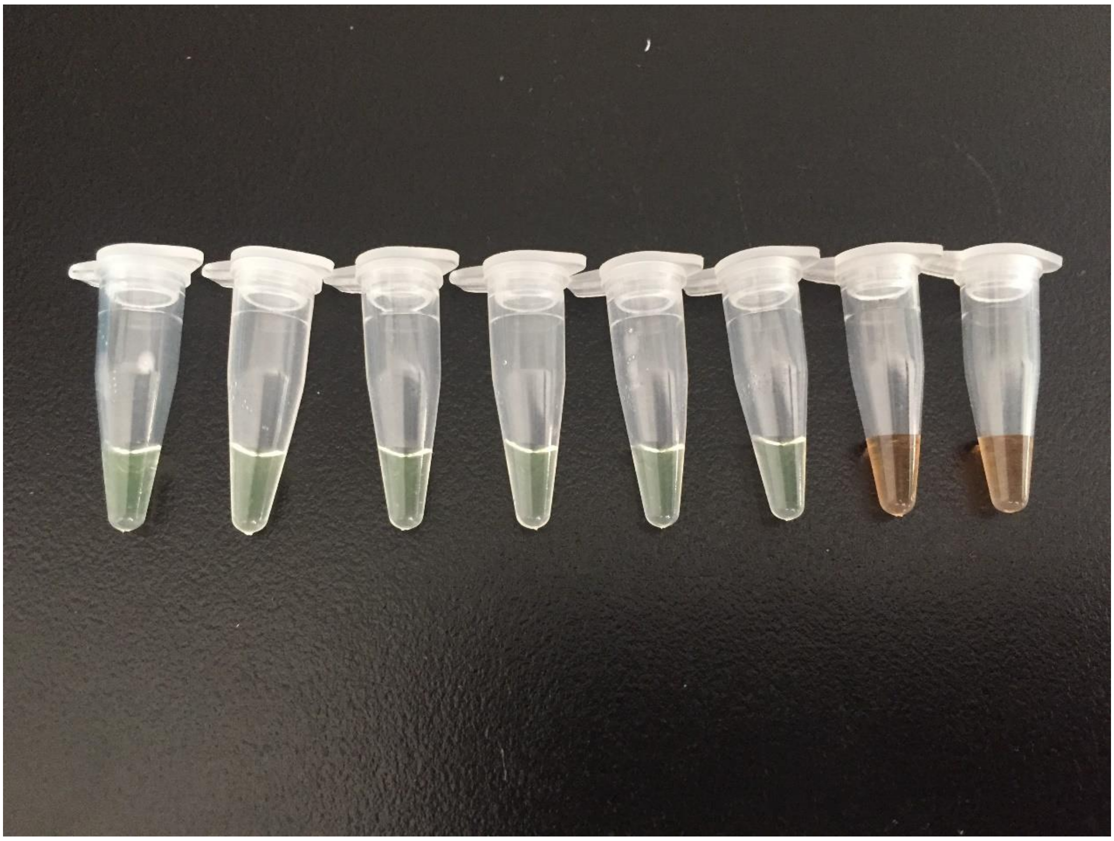

Supplementary Figure 3B

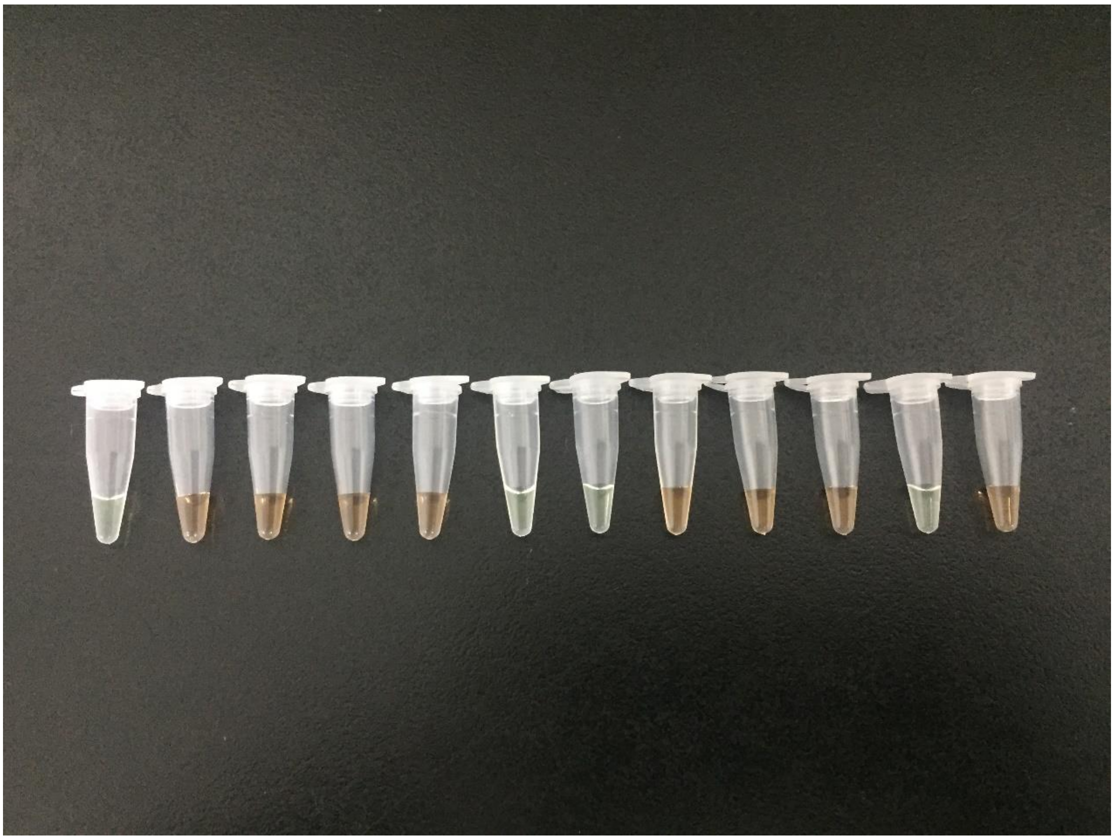

Supplementary Figure 4A

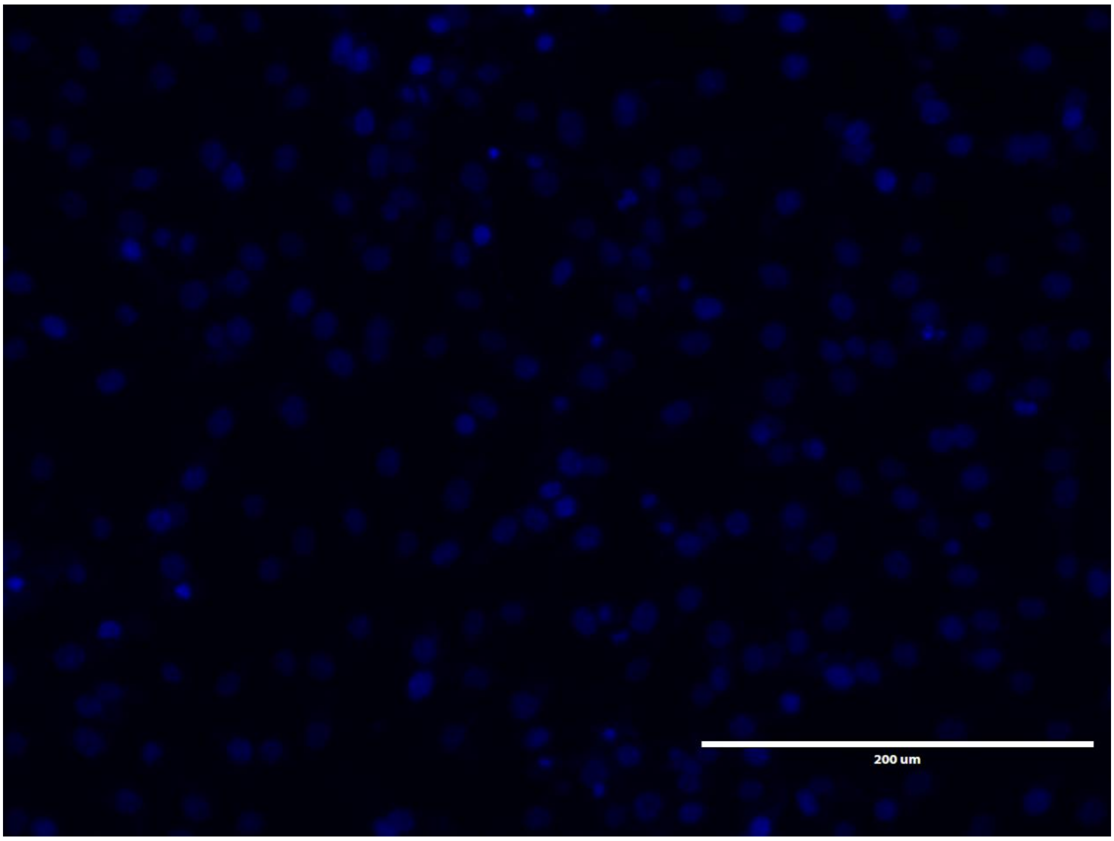

Supplementary Figure 4B DAPI1

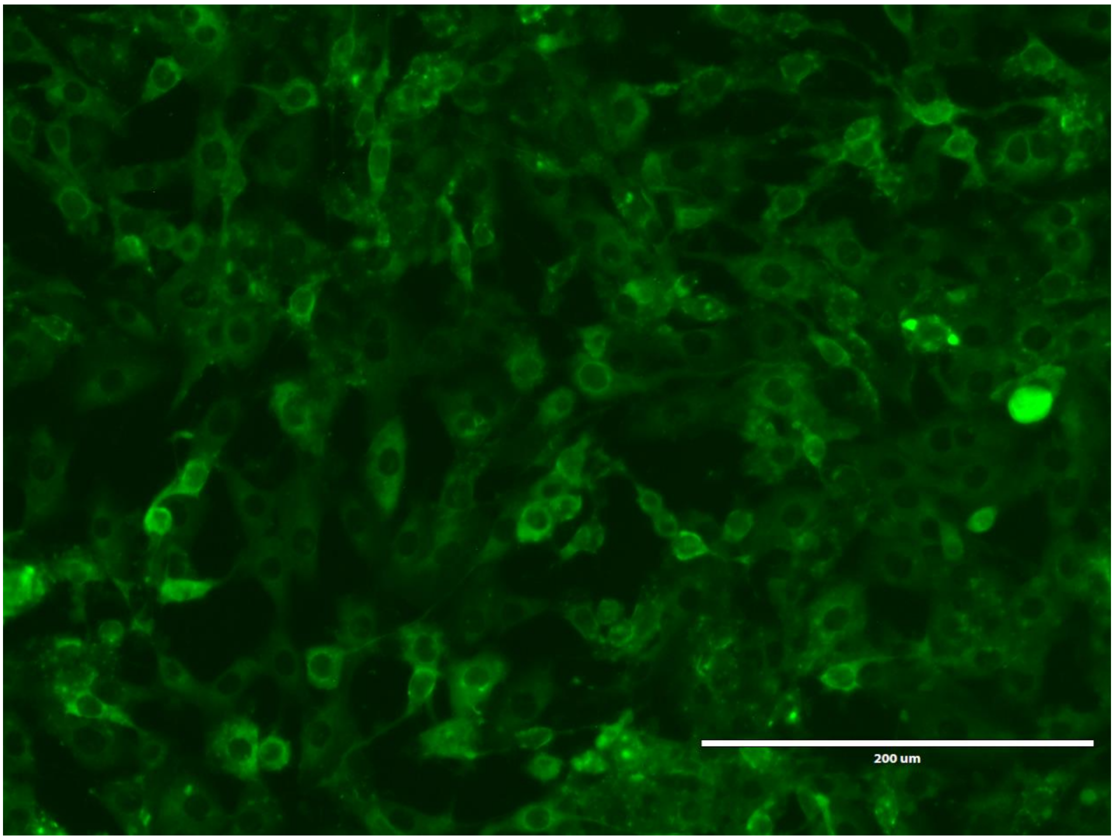

Supplementary Figure 4B FITC1

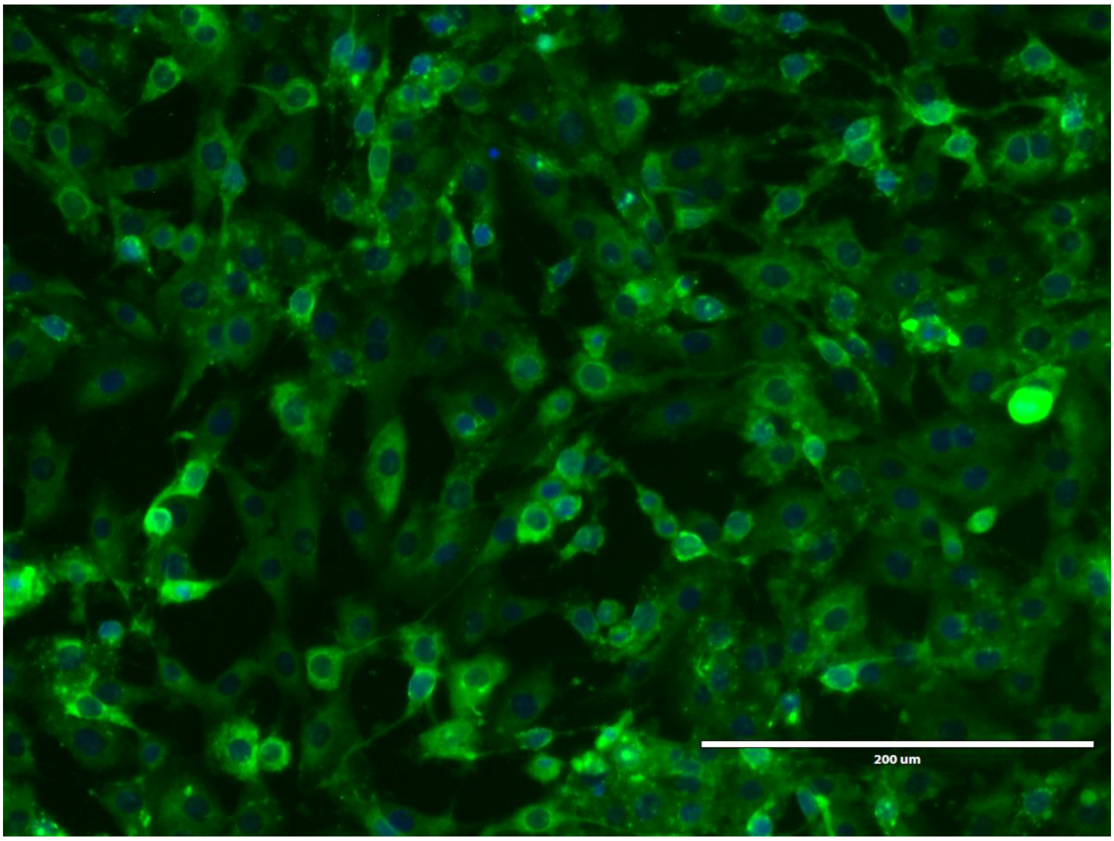

Supplementary Figure 4B Merge1

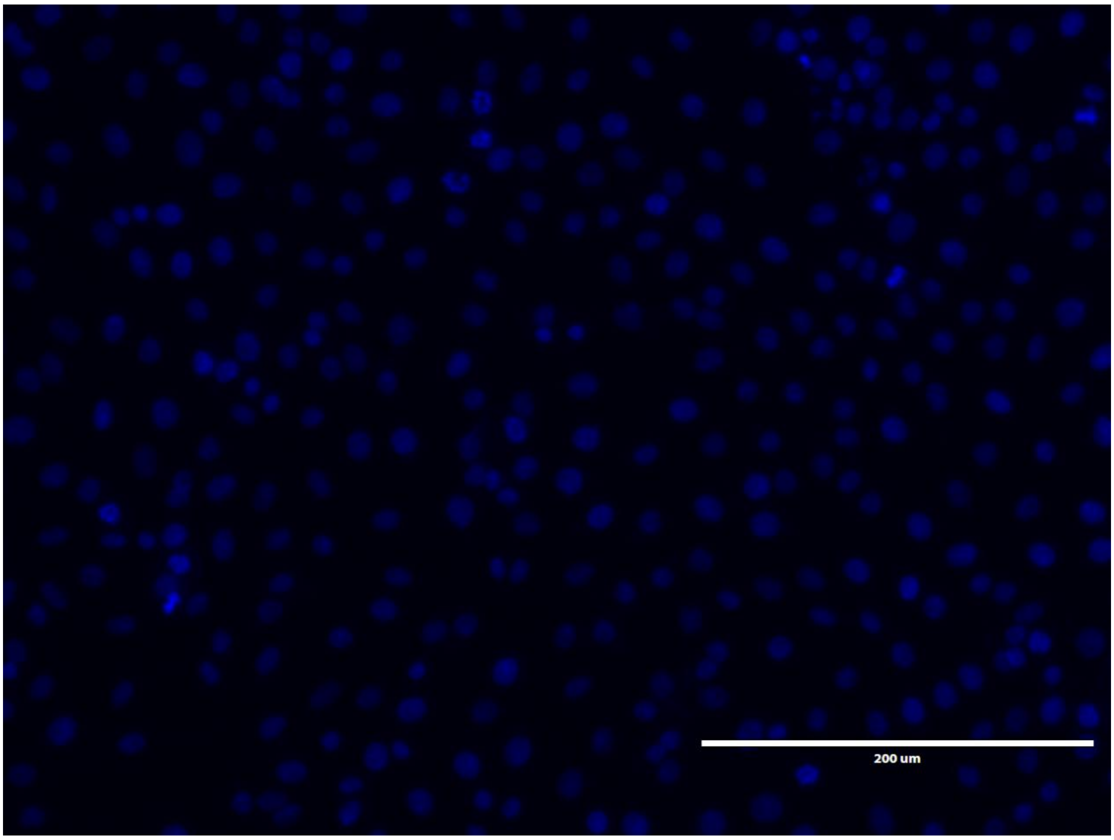

Supplementary Figure 4B DAPI2

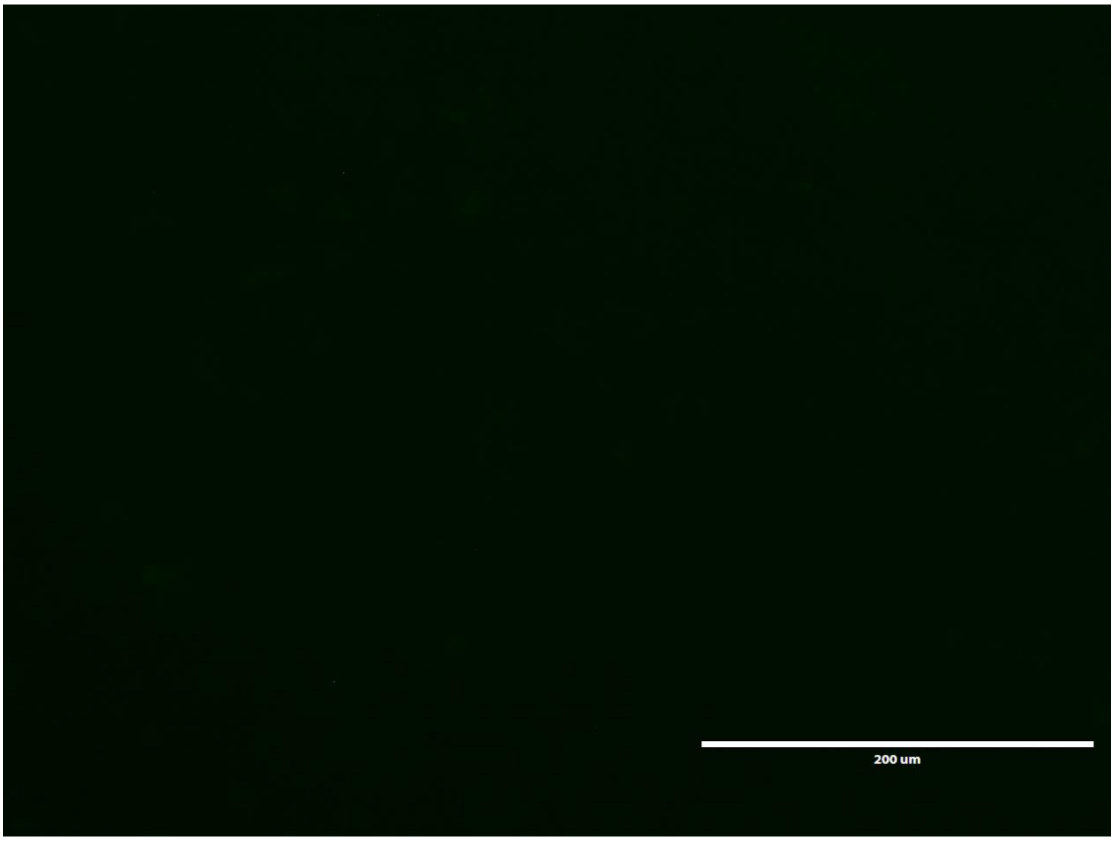

Supplementary Figure 4B FITC2

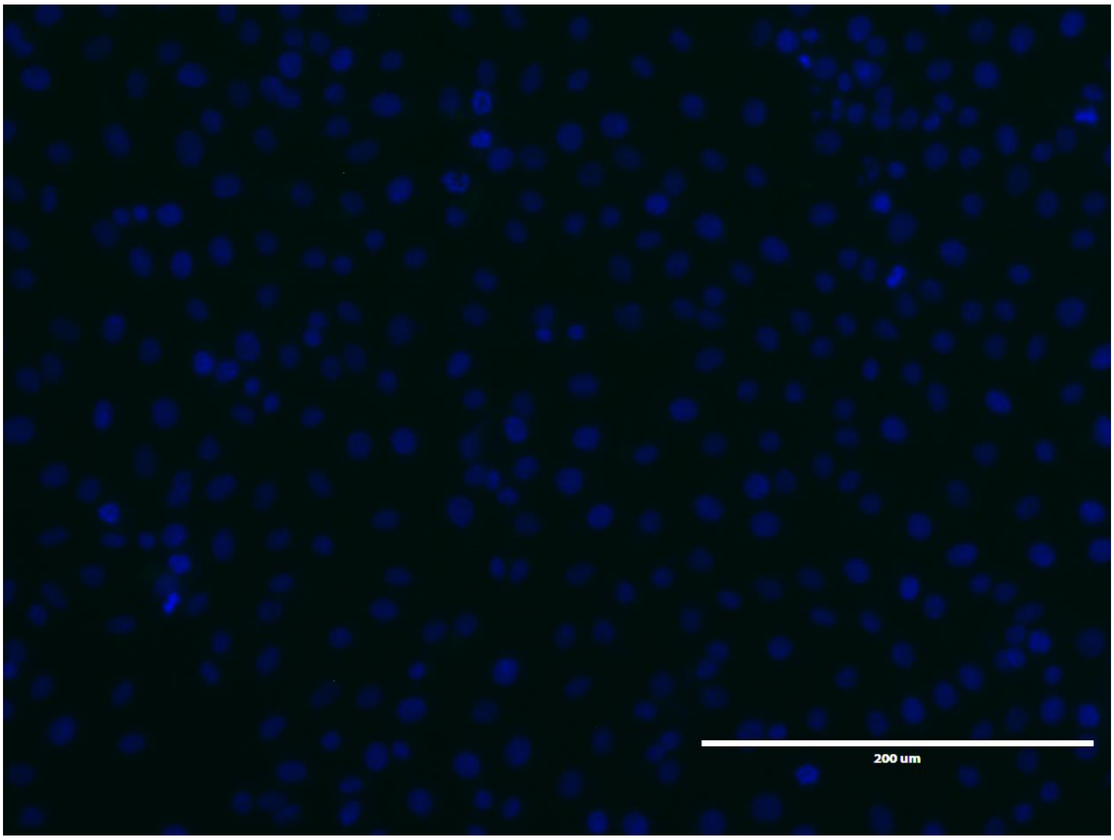

Supplementary Figure 4B Merge2
