# Supplementary material for: Loop-mediated Isothermal Amplification-Single Nucleotide Polymorphism Analysis for Detection and Differentiation of Wild-type and Vaccine Strains of Mink Enteritis Virus
Source: Sci Rep. 2018 May 30;8:8393. doi: 10.1038/s41598-018-26717-6 (PMC5976767; doi:10.1038/s41598-018-26717-6)
Supplement: Supplementary file 2 — Supplementary table 1 [file 41598_2018_26717_MOESM2_ESM.doc]

Loop-mediated Isothermal Amplification-Single Nucleotide Polymorphism Analysis for Detection and Differentiation of Wild-type and Vaccine Strains of Mink Entertis Virus

Peng Lin^1^, *, Honglin Wang^2^, *, Yuening Cheng^1^, Shanshan Song^1^, Yaru Sun^1^, Miao Zhang^1^, Li Guo^1^, Li Yi^1^, Mingwei Tong^1^, Zhigang Cao^1^, Shuang Li^1^, Shipeng Cheng^1^ & Jianke Wang^1^

^1^Key Laboratory of Special Animal Epidemic Disease, Ministry of Agriculture, P.R. China; Institute of Special Animal and Plant Sciences, Chinese Academy of Agricultural Sciences, Changchun 130112, People’s Republic of China

^2^Shandong Sinder Technology Co., Ltd，Zhucheng, Shandong 262204, People’s Republic of China

Key Laboratory of Special Animal Epidemic Disease, Ministry of Agriculture, P.R. China; Institute of Special Animal and Plant Sciences, Chinese Academy of Agricultural Sciences, No. 4899, Juye Street, Jingyue District, Changchun, People’s Republic of China 130112.

*These authors contributed equally to this work.

Tel.: +86 431 81919845; Fax: +86 431 81919800.

Correspondence and requests for materials should be addressed to J.-K.W. (email: tcswjk@126.com)

Supplementary Table 1

Detailed information of the 171 mink samples in the study

| Diarrhea | Yes | 99 |
| --- | --- | --- |
|  | No | 72 |
| Gender | Male | 91 |
|  | Female | 80 |
| Origin | Hebei | 20 |
|  | Shandong | 53 |
|  | Jilin | 29 |
|  | Liaoning | 42 |
|  | Heilongjiang | 27 |
| Age in months | <2 | 20 |
|  | 2-3 | 41 |
|  | 3-4 | 56 |
|  | 4-5 | 39 |
|  | >5 | 15 |
